# Supplementary material for: ALV-miRNA-p19-01 Promotes Viral Replication via Targeting Dual Specificity Phosphatase 6
Source: Viruses. 2022 Apr 13;14(4):805. doi: 10.3390/v14040805 (PMC9024826; doi:10.3390/v14040805)
Supplement: Supplementary file 1 [file viruses-14-00805-s001.zip › Supplementary Figure.pdf]

Figure S1

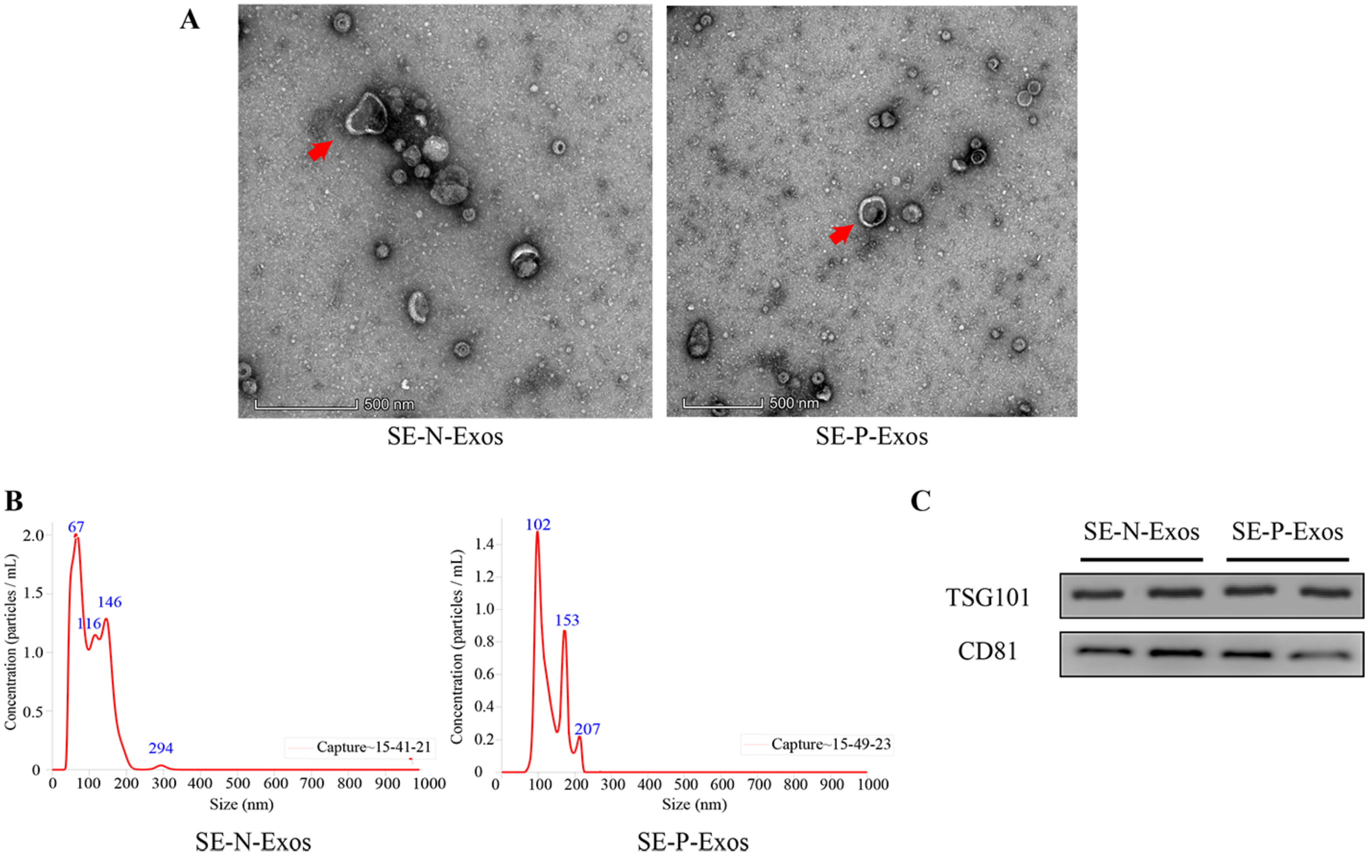

Figure.S1:Identification of semen exosomes from cocks. (A) Transmission electron microscope for observing morphology of exosomes. (B) Nanoparticle Tracking Analysis for analyzing SE-N-Exos and SE-P-Exos.(C) Western Blot analysis of the exosomal-marker proteins TSG101 and CD81. SE-N-Exos: semen exosomes of ALV-J-negative cocks, SE-P-Exos: semen exosomes of ALV-J-positive cocks.

Figure S2

pre-ALV-miRNA-p19-01		------------CCUGGGAUCCCAUUACCGCGGCACUCACCCAGCGGGCAAUGGUACUUGGGAAAT	**Strain**
ALV subtypes	K	CCGGGGTCCTGGGATCCCATTACCGCGGCACTCACCCAGCGGGCAATGGTACTTGGGAAAT	JS13LY19
	J	CCGGGGTCCTGGGATCCCATTACCGCGGCACTCACCCAGCGGGCAATGGTACTTGGGAAAT	NX0101
		CCGGGGTCCTGGGATCCCATTACCGCGGCGCTCTCCCAGCGGGCAATGGTACTTGGGAAAT	SCAU1903
	E	CCGGGGTCCTGGGATCCCATTACCGCGGCACTCACCCAGCGGGCAATGGTACTTGGGAAAT	AF229
		CCGGGGTCCTGGGATCCCATTACCGCGGCACTCACCCAGCGGGCAATGGTACTTGGGAAAT	ALVE-B9
	D	CCGGGGTCCTGGGATCCCATCACTGCGGCGCTCTCCCAGCGGGCAATGATACTTGGAAAAT	Schmidt-Ruppin D
	C	CCGGGGTCCTGGGATCCCATTACCGCGGCGCTATCCCAGCGGGCTATGATACTTGGGAAAT	Prague
	B	CCGGGGTCCTGGGATCCCATTACCGCGGCACTCACCCAGCGGGCAATGGTACTTGGGAAAT	GX14FF03
		CCGGGGTCCTGGGATCCCATTACCGCGGCACTCACCCAGCGGGCAATGGTACTTGGGAAAT	WD11150b
	A	CCGGGGTCCTGGGATCCCATTACTGCGGCACTCACCCAGCGGACAATGGTACTTGGGAAAT	SDAU09E1
		CCGGGGTCCTGGGATCCCATTACCGCGGCGCTCTCCCAGCGGGCTATGGTACTTGGGAAAT	SDAU09C3

Figure.S2: Conserved analysis of ALV-miRNA-p19-01 among different ALV subtypes.

Figure S3

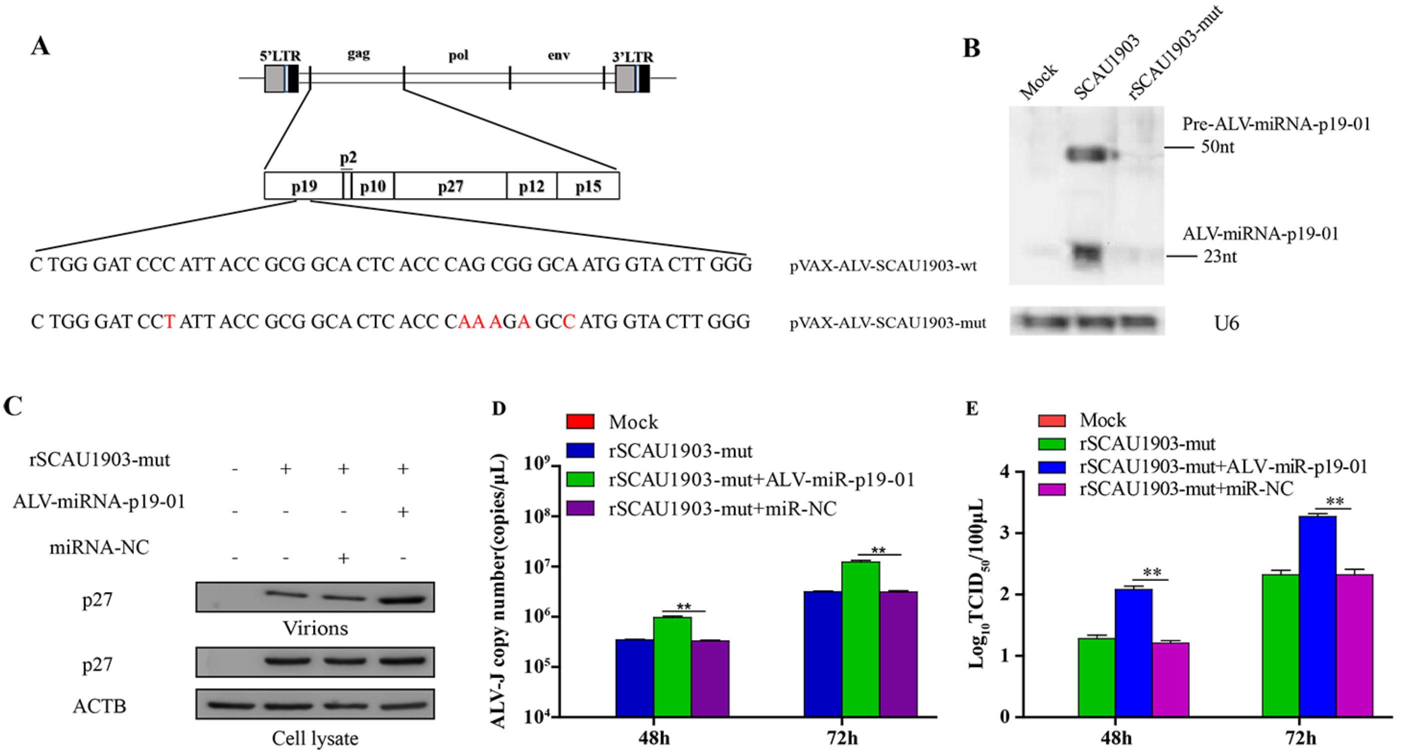

Figure.S3:ALV-miRNA-p19-01 promotes rSCAU1903-mut release. (A) Mutant sequence of pVAX-ALV-SCAU1903-mut compared to that of pVAX-ALV-SCAU1903-wt. (B) DF-1 cells were infected with ALV-J SCAU1903 or rSCAU1903-mut. The RNA was used to detect the ALV-miRNA-p19-01 by Northern Blot, U6 as a control. (C to E) DF-1 cells respectively transfected with ALV-miRNA-p19-01 mimic and miR-NC were infected with rSCAU1903-mut at 4 hpt. (C) The expression of p27 protein were examined by Western Blot. (D) The transcriptional level of viral particles in supernatants were measured by RT-PCR. (E) The supernatants from infected cells were titrated in DF-1 cells by ELISA for $TCID_{50}$.

Figure S4

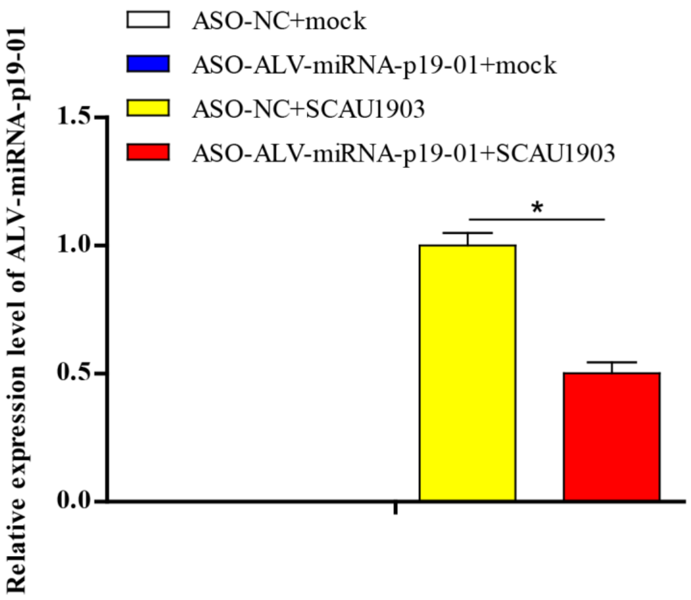

Figure.S4: The inhibitory efficiency of ASO-ALV-miRNA-p19-01. ASO-ALV-miRNA-p19-01 was transfected into DF-1 cells and then infected with ALV-J SCAU1903 at 4 hpt. ALV-miRNA-p19-01 expression was examined by RT-qPCR.

Figure S5

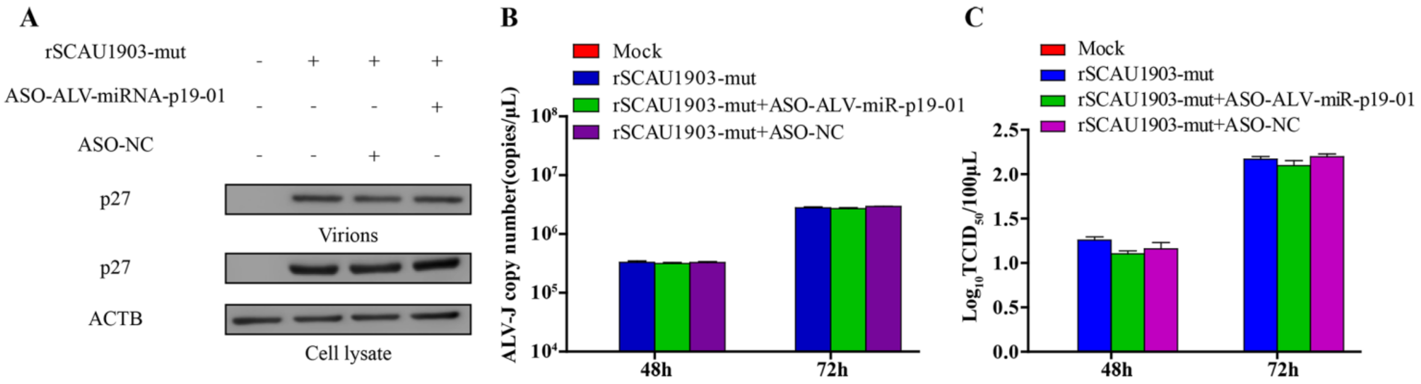

Figure.S5:ASO-ALV-miRNA-p19-01 does not affect rSCAU1903-mut release. (A to C) DF-1 cells respectively transfected with ASO-ALV-miRNA-p19-01 mimic and ASO-NC were infected with rSCAU1903-mut at 4 hpt. (A) The expression of p27 protein were examined by Western Blot. (B) The transcriptional level of viral particles in supernatants were measured by RT-PCR. (C) The supernatants from infected cells were titrated in DF-1 cells by ELISA for $TCID_{50}$.

Figure S6

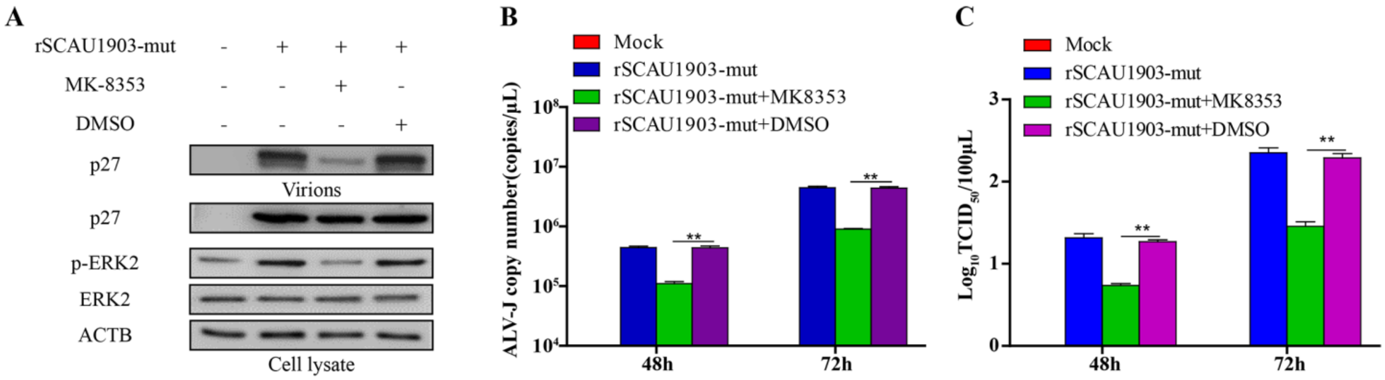

Fig.S6: Effect of ERK2 phosphatase activity on ALV-J release. (A to C) DF-1 cells were respectively treated with MK-8353 and DMSO for 1 h, and then DF-1 cells were infected with rSCAU1903-mut. (A) The expression of p27 protein were examined by Western Blot. (B) The transcriptional level of viral particles in supernatants were measured by RT-PCR. (C) The supernatants from infected cells were titrated in DF-1 cells by ELISA for $TCID_{50}$.
